# Havening: a psycho-sensory therapy for enhancing emotional resilience and psycho-emotional wellbeing across the perinatal period

**DOI:** 10.3389/fgwh.2025.1619273

**Published:** 2025-07-08

**Authors:** Susan Crowther, Christine Mellor, Kimm Sun

**Affiliations:** ^1^ AUT University Faculty of Health and Environmental Sciences, Auckland University of Technology, Auckland, New Zealand; ^2^ Sunrising Midwifery New York, New York, NY, United States

**Keywords:** psycho-sensory, havening, perinatal mental health, childbirth, trauma

## Abstract

Havening, a psycho-sensory therapy, is increasingly recognized for its potential in supporting perinatal psycho-spiritual and emotional health. By employing gentle touch and guided imagery, Havening aims to reduce distressing emotions and enhance well-being. This article explores its application on emotional processing in perinatal care, with a particular focus on its impact on the amygdala, the brain's emotional processing center. The theoretical foundations proposed by Dr. Ronald Ruden regarding Havening's neurobiological effects are discussed, followed by the presentation of four case studies and respective outcomes that demonstrate the potential of Havening in perinatal mental health.

## 1 Introduction

The perinatal period, which consists of pregnancy and up to one year postpartum, is a time of significant psychological, social, and emotional transformation (1–5). Women are more likely to develop a mental health disorder during this period than at any other time in their lives, which can have significant associated morbidities for the woman, baby, and family (6). Many women experience heightened vulnerability to stress, anxiety, and trauma-related responses across the childbirth year (7). The global prevalence of perinatal mental health disorders is concerning (8). The WHO estimates 10%–13% of women will develop mental health disorders postnatally (9), which is considerably higher in LMIC countries (10). However, a wide regional variance is acknowledged in these estimates due to reporting, health service infrastructure and socio-cultural contexts. For example, an estimated ten to twenty percent of birthing women/parents in Aotearoa New Zealand, a bicultural country, experience perinatal distress that is significant enough to meet clinical mental health definitions (11). Investing in emotional wellbeing during this period is therefore critically important. Yet equity of investment in this area is not evident globally (8). In Aotearoa New Zealand, where two of the authors reside, there is a “disparity between needs and service provision (12 p.30)” in relation to women with perinatal mental health distress considered to be mild/moderate in nature and appropriate maternal mental health services.

Maternal brain plasticity, including changes in the maternal amygdala, have been noted in the postpartum period (13–15). For example, during pregnancy and postpartum, hormonal shifts increase amygdala sensitivity, contributing to heightened emotional reactivity and susceptibility to stress (16). This dysregulation of the amygdala is being linked to conditions such as perinatal anxiety, depression, and post-traumatic stress disorder (PTSD) (17). Furthermore, functional impacts on the neonate have been noted in the context of maternal stress and anxiety (18, 19). Significantly, studies looking at maternal mental health provide evidence for subtle but long-lasting alterations to amygdala morphology associated with differences in maternal anxiety in early development of offspring (20–22). This could be linked to the intrauterine environment and the etiology of neuropsychiatric disorders in offspring (23). This highlights the importance of attending to maternal amygdala health in the perinatal period because of potential intergenerational impact of maternal stress, fear and trauma on the next generation's amygdales (24). Although some of this neurobiological science is still theoretical, there is an emerging understanding about the centrality of amygdala health.

Whilst there are psychological and pharmacological treatments that have been shown to be effective in treating perinatal distress (25), many women prefer a non-medicated approach due to concerns about medication transmission to their baby during pregnancy or lactation (26). In this article, we consider an innovative approach, called Havening Techniques (HT), that is theoretically based on neurobiological mechanisms. Case studies are presented that highlight the potential positive impact of HT on women experiencing mild to moderate perinatal mental distress. Havening Techniques, a relatively new therapeutic approach, integrates touch with cognitive interventions to gently and effectively regulate emotional responses (27). Havening Techniques comes under the umbrella of psychosensory therapies (2) that do not use any pharmaceutical agents and are distinct from talk therapies like counseling and psychotherapy where clients need to talk through their concerns with a therapist. On the contrary, HT does not require the client to repeatedly talk about their trauma and distress (14). Thus, HT provides a non-pharmacological alternative, or an alongside modality to these therapies, broadening the scope of available services and increasing choice for women and families.

**Figure 1:**
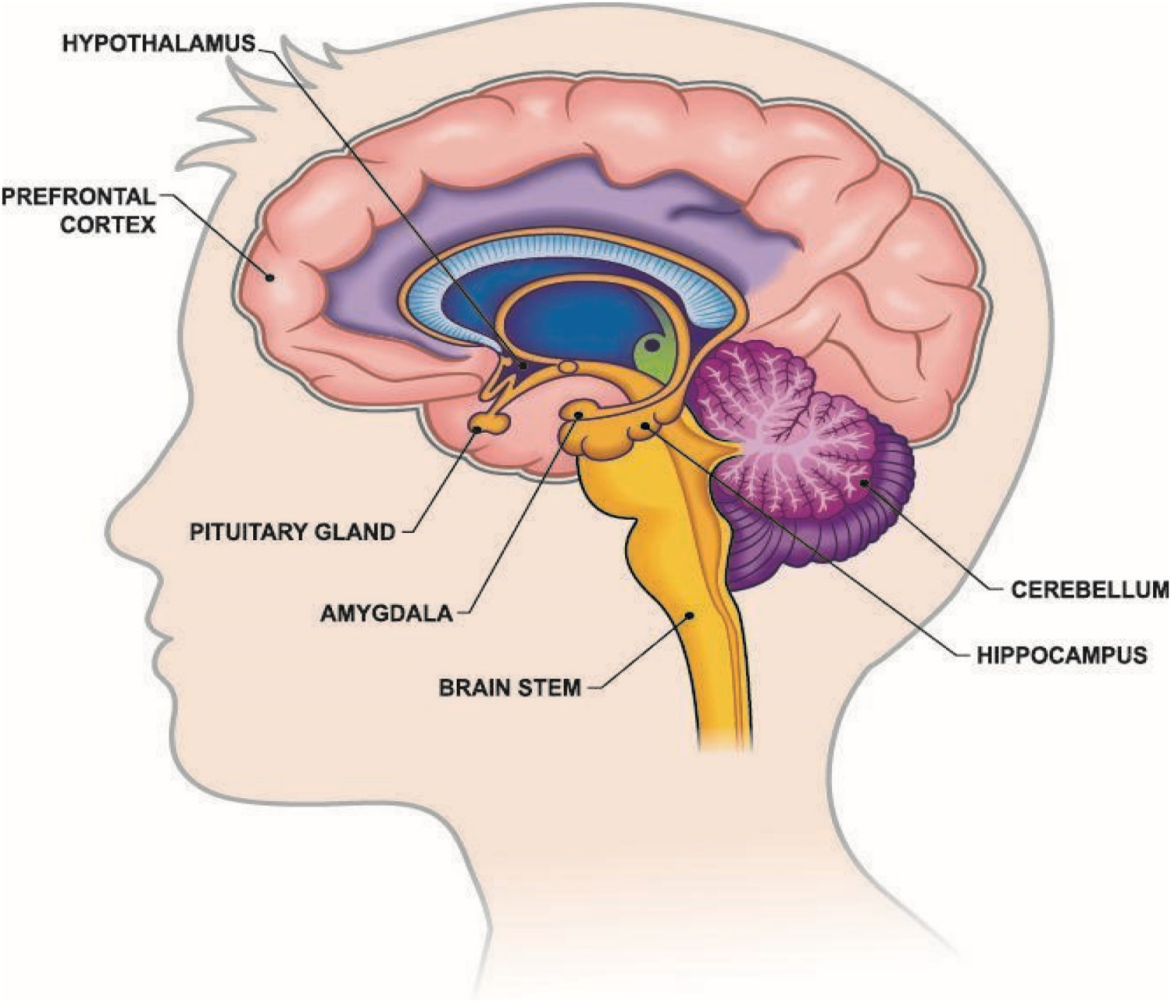
Brain anatomy showing location of the Amygdala: image public 25/3/25 IStockPhoto.

According to Ruden's theoretical reasoning Havening Techniques (HT) has a direct physiological effect on the brain, specifically the amygdala (Figure 1) (14). However, this direct effect on the amygdala requires further empirical examination. What is empirically established is that the amygdala is a key region involved in emotional memory and threat perception and is central to processing emotions, particularly fear and stress responses (29–31), and that responses are individually unique (32). There is an emergent body of empirical work being carried out into the effectiveness of HT. Interestingly, there is some evidence that personality types, specifically type D personality, may be more sensitive and impacted by HT (33). Likewise, the effectiveness of HT for physical pain, specifically surgical pain, has been explored although this remains inconclusive (34).

The proposed theoretical considerations provided by Ruden's work, and the underlying neuroscience about the functions of the amygdala, provide insight into how HT's therapeutic benefits for perinatal mental health could be possible. Dr. Ronald Ruden, the creator of HT, posits that traumatic memories become encoded in the amygdala through heightened electrochemical activity, and that HT can depotentiate these pathways, reducing emotional distress (14). In this article we present a theoretical proposition that hypothesizes that HT, a psycho-sensory therapy, has a place in the lexicon of helpful approaches in perinatal psycho-emotional wellbeing, and deserves further examination.

The first part of this article describes what Havening is and what HTs are, including the bioelectrical-biochemical processes, and the actions of potentiation and depotentiation in the amygdala. The types of techniques are then described along with a typical HT session. Four case studies are then presented to demonstrate the impact that HT can have on perinatal mental health. This is not a formal research project, and women's case studies are drawn from previous practice experiences. All women have agreed and consented, prior to this article, to their stories being used anonymously as case studies for this article. The case studies are purely illustrative of HT potential in this domain and are not formally analyzed.

## 2 What is havening?

To understand the mechanisms of HTs we begin with an explanation of how traumatisation becomes encoded and potentiated in the body (1). It is hypothesized that traumatization occurs when four specific requirements for encoding trauma are present. In Table 1 a consolidated synopsis of these requirements and components are provided and are then referred to in the four case studies described and discussed in section 7.

**Table 1:** Requirements of encoding and components of traumatization [consolidated from ruden (32)].

Requirements of encoding: EMLI		Components of traumatic memory: CASE	
Event	The traumatic event e.g., difficult out of control birth experience. (Actual or perceived)	Cognitive component	Non-emotional content of traumatic memory, may include adverse childhood events (ACE), e.g., an unspecified threat stimulus (UTS) such as being abandoned
Meaning	The event generates an emotional response, e.g., fear.	Autonomic reactions	e.g., shaking, sweating, blushing,
Landscape	History of current and life circumstances, level of resilience, coping; vulnerability, e.g., in marriage breakup at time of a birth trauma	Somatosensory aspects	e.g., pain, headache,
Inescapability (perceived or actual)	Unable to get away e.g., feeling trapped on the hospital bed in stirrups	Emotional content	e.g., fear, terror, confusion, loneliness

**Figure 2:**
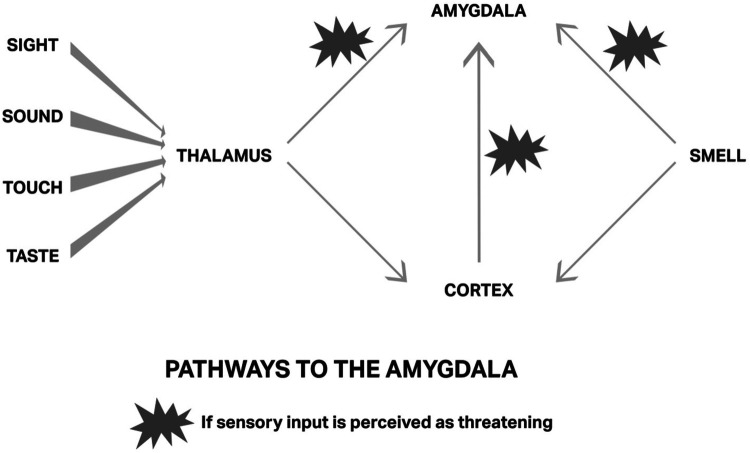
Pathways through the Amygdala: taken with permission from Ruden, Primer (32).

When a traumatic or distressing experience occurs, the amygdala encodes the memory by laying down new synaptic connections through a process called potentiation. This occurs via a surge of calcium ions into neurons, activating NMDA receptors and reinforcing the neural circuitry associated with the traumatic event. This potentiation results in the persistent reactivation of distressing emotions when triggered by similar stimuli in the future. This involves pathways to the amygdala shown in Figure 2, illustrating the sensory input required for the encoding of trauma to occur.

Havening Touch is believed to release the established specific neural connections to the traumatic memory; in other words, de-encode the memory (32). By permanently delinking the amygdala to negative emotional responses, HT allows individuals to process perinatal challenges with greater resilience and emotional balance. For example, imagine a woman is experiencing a massive postpartum bleed; she “sees” the blood on the bed, floor and midwife's uniform. She hears the emergency buzzer calling for help, her newborn crying, noise of metal instruments and people entering the room talking quickly and loudly. She feels the physical touch of others as they insert needles in her arm quickly and attach a blood pressure cuff and suddenly notices a metallic and bitter taste developing in her already dry mouth. She is also aware of smelling antiseptics, toast, coffee, and body odors. She feels trapped, alone, exhausted, terrified and believes she will die. It can be construed in this scenario that she is experiencing all the requirements of encoding trauma, and all four components for encoding a traumatic memory (Table 1). She may be undergoing a potentiating event, perhaps leaving her triggered each time she smells coffee or sees blood. This of course would also be dependent on the landscape of her brain (level of vulnerability/resilience because of past experiences) which influences how present experiences are perceived and how traumatizing they may be to the individual. Likewise, previous related or unrelated traumatic experiences tend to be a predisposing factor to further traumatization (14).

Havening Touch involves the application of slow, rhythmic touch on the face, arms, and hands while engaging in guided mental exercises. The skin has receptors called C-tactile fibers, a class of unmyelinated, mechanosensitive nerve fibre (36, 37). These fibers can transmit sensory information from touch on the skin to the brain. There are different ways of experiencing touch: e.g., fast touch, harsh touch, pinch, and slow, gentle and nurturing touch. Havening Touch employs a soothing comfortable touch akin to a tear being wiped away. This quality of gentle touch transmits information up to the insula, a region of the brain which has a crucial role in various functions, e.g., sensory processing, emotional regulation, and decision-making, as well as influencing different areas in our prefrontal cortex to shift how the brain interacts with the present moment.

It is known that social touch stimulates the release of serotonin and oxytocin, which counteract stress hormones like cortisol (38). Repetitive touch applied in HT sessions is proposed to have a similar effect. Oxytocin, a nanopeptide that has several functions throughout the brain and body, is associated with a sense of safety and psychosocial connectedness (39). Moreover, nurturing touch can also reduce blood pressure, and enhance heart rate variability (40). Touch performed in the right manner creates connection to and engagement with self and others (41). The type and quality of touch is a crucial element in HT. Empirical work focusing on touch in HT found that a downward change in SUDs and change in brain function, measured shortly after the session, occurred within a single Havening session (42). Further analysis of Sumich's research suggests a reduction in anterior temporal lobe activity following a single 20-min session of HT (in Press, 2025). Whilst Sumich acknowledges that the EEG methods used in the study do not directly assess the amygdala function, they suggest that the reduction in anterior temporal lobe activity reflects activity downstream from the amygdala.

Ruden coins the term “electroceuticals”, which refers to various biological processes proposed to mitigate the stress response and disconnect the distressing memory from its physiologically encoded components. That is, dissociating the memory from the individual's physiological/emotional response to it (2). In the following section, three key concepts in HT are unpacked related to delta wave electrical activity, biochemical processes, and depotentiation.

## 3 Delta wave electrical activity, biochemical processes, and depotentiation

Evidence from neuroscience suggests that the electrical activity in the body, or oscillatory activity, is connected with a variety of perceptual, sensorimotor, and cognitive processes (43). For example, there are suggestions that delta oscillations in memory reactivation occur, although this requires further empirical work to verify (44). These slow-frequency waves (0.5–4 Hz) are typically predominant during deep non-REM sleep but can also be induced during certain meditative and therapeutic states (45, 46), and potentially occur in a session of HT. However, we need to be cautious in making proven associations and acknowledge this is a theoretical proposition requiring further empirical examination. [For a more nuanced neuroscience review on Delta oscillations/waves see a review by Knyazec (42)].

**Figure 3:**
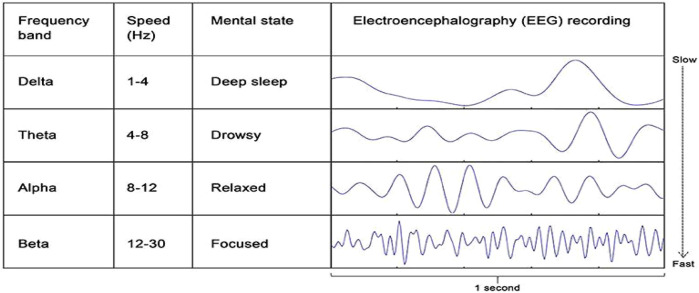
Brain waves: https://kids.frontiersin.org/articles/10.3389/frym.2020.00096 accessed 25/3/25.

Figure 3 shows the different brain waves or oscillations in the delta frequency, and how Delta waves induce a profound relaxed state akin to deep sleep.

The key effects of these Delta waves are summarized in Table 2.

**Table 2:** Four key effects of delta waves.

Delta waves	Effect
Emotional regulation	Delta waves are associated with reduced activity in the amygdala, promoting a state of calm and decreasing the intensity of emotional reactivity.
Neurochemical modulation	Delta wave activity increases the release of serotonin and oxytocin, which counteract stress hormones like cortisol and norepinephrine, enhancing emotional stability.
Memory reconsolidation and depotentiation	The presence of delta waves facilitates synaptic depotentiation, a process in which AMPA receptors are removed from synaptic connections, weakening the neural pathways that encode distressing memories. The AMPA receptor or AMPAR is an ionotropic glutamate receptor (iGluR) mediating excitatory neurotransmission in the central nervous system (CNS).
Increased neuroplasticity	By inducing a relaxed, receptive state, delta waves promote neuroplastic changes, allowing the brain to reprocess past experiences in a less distressing way.

In the context of HT, delta waves play a theoretically crucial role in emotional processing and neural depotentiation. In the context of perinatal mental health, delta waves generated through HT may help reduce anxiety, process birth trauma, and enhance maternal well-being by fostering a deeper sense of relaxation and emotional balance; in effect improving the person's landscape, that is, increasing resilience and capacity to meet the stressors of the world without or at least minimally being triggered.

When a distressing or traumatic event occurs, synaptic potentiation takes place in the amygdala, strengthening neural pathways that reinforce the emotional intensity of the memory. Table 3 shows the key electrical, biochemical and hormonal processes involved in potentiation, and highlights how a traumatic encoding event produces an actual physiological alteration in our neurological structures permanently. The original event, such as the woman described previously who experienced the massive postpartum haemorrhage, would, according to Ruden, become permanently encoded through synaptically connecting any of the four components. Ruden (14) explains how any one of the components of a traumatizing memory when recalled, consciously or unconsciously, can cause an individual to re-experience the traumatic event as if it was occurring for the first time.

**Table 3:** Electrical, biochemical and hormonal processes involved in potentiation.

Potentiation process	Action of the processes
Calcium influx	In response to emotional distress, calcium ions enter neurons through NMDA receptors, initiating intracellular signaling cascades.
AMPA receptor upregulation	The presence of calcium activates protein kinases, leading to the recruitment of additional AMPA receptors to the synaptic membrane. This strengthens the synapse, making it more responsive to future triggers.
Neurotransmitter release	Glutamate, the primary excitatory neurotransmitter, is released in greater quantities, further reinforcing synaptic strength and increasing emotional reactivity.
Stress hormones	Cortisol and norepinephrine enhance memory encoding, solidifying the distressing experience in long-term storage.

Initiation of a potentiation process can therefore have short- and long-term consequences for women experiencing traumatic childbirth events. Yet this process can be reversed through HT enabling depotentiation. Ruden describes depotentiation as the key mechanism by which HT facilitates emotional healing (14, 32). HT, Ruden theorizes, disrupts this process of potentiation by generating oscillations in the delta frequency, triggering a neurochemical shift that ultimately reduces the excitability and strength of these synaptic connections that maintain distressing emotional memories. Ruden suggests that this cascade of events causes depotentiation of the AMPA receptors at the synaptic membrane, which are activated when the distressing memory is recalled. It is theorized that an enzyme called calcineurin removes the phosphorous molecule that anchors the AMPA receptors, and the receptors are then removed from the surface membrane by a process of endocytosis (1).

Gentle touch used in HT also sends signals to the brainstem, promoting the release of serotonin and oxytocin, counteracting stress responses. GABA, a primary inhibitory neurotransmitter, also has a calming influence on the nervous system that helps reduce anxiety (47). This effect may lead to lowering stress by potentially desensitizing traumatic memories, creating a sense of well-being and inducing relaxation.

In sum, Ruden suggests that the Havening touch signals safety, and the cascade of events at the synapse effectively weakens the encoded traumatic memory. Theoretically, this depotentiation, and the emotional charge associated with distressing memories is reduced, allowing individuals to recall past experiences without experiencing the same level of stress, fear, anxiety, or anger (2). Further empirical studies are required to confirm these physiological effects in HT.

In the context of perinatal mental health, it is presently proposed that delta waves activated through HT would help reduce anxiety, process birth trauma, and enhance maternal well-being by fostering a deeper sense of relaxation and emotional balance. This would be expected to strengthen resilience and capacity to meet the stressors of the world, whilst reducing the risk of being triggered during therapy. Returning to the woman who experienced childbirth trauma who is subsequently triggered into emotionally charged responses to the smell of coffee and blood, those emotional responses can be fully or partially diminished through the depotentiation of the AMPA receptors. This does not mean that the recall memory is removed. After the depotentiation, the recall is no longer accompanied by an emotionally charged response.

The following five steps summarize the theoretical impact of HT:

1. Delta Wave Induction: The gentle touch and rhythmic nature of Havening generates oscillations in the delta frequency, which have a calming effect on the nervous system.
2. AMPA receptor Removal/depotentiation: Delta waves trigger the internalization of AMPA receptors, reducing synaptic responsiveness to stress-related triggers.
3. GABAergic Modulation: The production of gamma-aminobutyric acid (GABA) is increased, inhibiting excessive excitatory signaling and restoring emotional balance.
4. Serotonin and Oxytocin Release: These neurochemicals promote relaxation, bonding, and resilience, counteracting the effects of cortisol and norepinephrine.
5. Electrophysiological Stabilization: By reducing hyperactive electrical activity in the amygdala, Havening helps shift neural networks toward a more adaptive state, preventing automatic emotional reactivation.

These biochemical and electrical changes may explain why individuals who receive HT often report feeling detached from previously distressing memories, experiencing them as less emotionally charged. In the perinatal context, this mechanism has been observed by midwives trained in HT who apply this modality to help alleviate trauma associated with childbirth, postpartum distress, or perinatal anxiety, exemplified through four case studies in section 7.

### 3.1 Havening techniques sessions

The HT practitioner works with five basic Havening techniques (see Table 4). There are other techniques used, but these are the foundational ones. Several techniques can be used in a single session, according to the situation.

**Table 4:** Five foundational havening techniques.

Type of havening technique	Therapeutic description
EH - Event Havening	This is the main technique at the core of Havening techniques (HT). In this technique the client is asked to recall a distressing event and provide SUDs (see Table 1: Abbreviations and acronyms). HT is applied while distractions are used. Distractions are simple mental tasks such as counting, humming, naming different countries, etc.
TH - Transpiration Havening	Clients are asked to identify difficult emotions, such as anger, guilt, or abandonment. Each emotion is repeated as touch is applied until the emotion is no longer felt or has shifted to a different emotion. TH is repeated until the identified emotions have been released, i.e., transpired. TH is not recommended during pregnancy due to the potential for emotional flooding. Instead, a hybrid of TH/EH is used. Once the emotion is identified, a SUDs is taken, and distractions are used. TTH - Talk Transpirational Havening, where client is speaking freely while HT is applied, is a gentler version that is less likely to trigger emotional flooding.
OH – Outcome Havening	Clients are asked to imagine a different outcome from the traumatic event while HT is applied. For example, if the event happened during childhood, the client could be asked: “Can an adult come and save you from this situation?” Likewise, this can be used for future events such as preparation regarding fear of going to a hospital appointment.
RH – Role Havening	The Haveners take on the role of the “antagonist” and provide the opportunity for the client to have a conversation with said antagonist. For example, the Havener would invite the client to speak to an abusive or neglectful parent and express feelings that would otherwise not be possible. They can also ask for the abuser to say something specific in response.
AF - Affirmational Havening	Normally used once the SUDs are three or less. Clients are asked for their own affirmations, such as “Calm,” “Light,” “Peaceful,” or “Hopeful."

There are other techniques too depending on client needs. For example, Metaphorical Havening, Photostat, Mantra Havening, and Colour Havening.

**Table 5:** Abbreviations and acronyms.

Abbreviations and Acronyms	Description and meaning
SUDs	Subjective Units of Distress Scale
CASE	Cognitive, Autonomic, Somatosensory, Emotions
EMLI	Event, Meaning, Landscape, Inescapability
ACEs	Adverse Childhood Events
HT	Havening Techniques
GABA	Gamma-aminobutyric acid (an amino acid)
AMPA(R)	*α* -amino-3-hydroxy-5-methyl-4-isoxazolepropionic acid (receptor)
NMDA	N-methyl-D-aspartate: a type of glutamate receptor, a ligand-gated ion channel in the brain activated by the neurotransmitter glutamate
G (number) P (number)	Gravida (number of pregnancies) Parity (number of births)

Havening Techniques sessions are usually 60–90 min in length, with the Havening touch typically lasting 20–40 min. Sessions begin with time for connection between the woman and the Havening practitioner, laying foundations for feeling safe and calm. The science behind HT and the process are both fully explained, and if the woman feels able, she shares the experience, emotion, or self-belief that she would like to resolve. The woman is then guided to express what her needs are, and how the trauma has manifested; cognitive, autonomic, somatosensory, and emotional effects are explored. A careful history is essential, as there may be other life experiences or events that are significant; this makes up what is called the landscape of the client in HT. For example, the woman may describe a fear of giving birth, but beneath this may be first-hand experience of a traumatic birth, a traumatic birth that she has been present at, or/and a significant life event where she felt vulnerable, exposed, or under threat. It is important to note that the benefits of HT still occur even if a traumatic event is not shared verbally in detail with the Havening practitioner; the event only requires it to be recalled. The Havening practitioner decides which of the Havening Techniques would be most appropriate to start with, and sometimes more than one technique is used.

To illustrate, one of the five foundations techniques, Event Havening (EH), is described here within a session. The woman and practitioner sit facing each other and alongside, having ascertained whether the practitioner will apply the Havening touch, or the woman would prefer to do this herself. The woman closes her eyes and for a moment, recalls the part of the memory that represents the peak of her distress. As mentioned previously she may describe verbally, or not, the memory of the event. She is asked to remember what she saw, heard, smelt, and felt, recalling all the sensory information, and to report a Subjective Unit of Distress (SUD) score where zero reflects calm and no distress, and ten represents extreme distress. The woman then clears her mind of these thoughts, and Havening Touch begins, alongside distractions.

Distractions are used to interrupt the continuous activation of the amygdala, preventing distressing thoughts from persistently resurfacing in the session by replacing the working memory (located in the prefrontal cortex) with simple mental tasks. Distractions can be cognitive, visual or auditory, and are often light-hearted and playful, for example, naming as many dog breeds as she can, counting backwards in 3s from 30, describing the stages in making a cake, or humming a tune, spelling a word backwards or walking on a beach counting each step. These mental distractions steer the woman away from thinking about the distressing memories, emotions and beliefs whilst the electrochemical process takes place to delink the neural pathway towards a fight or flight, or other sympathetic nervous system response. The overall aim of the session is to depotentiate the AMPA receptors in the Amygdala as described above.

**Figure 4:**
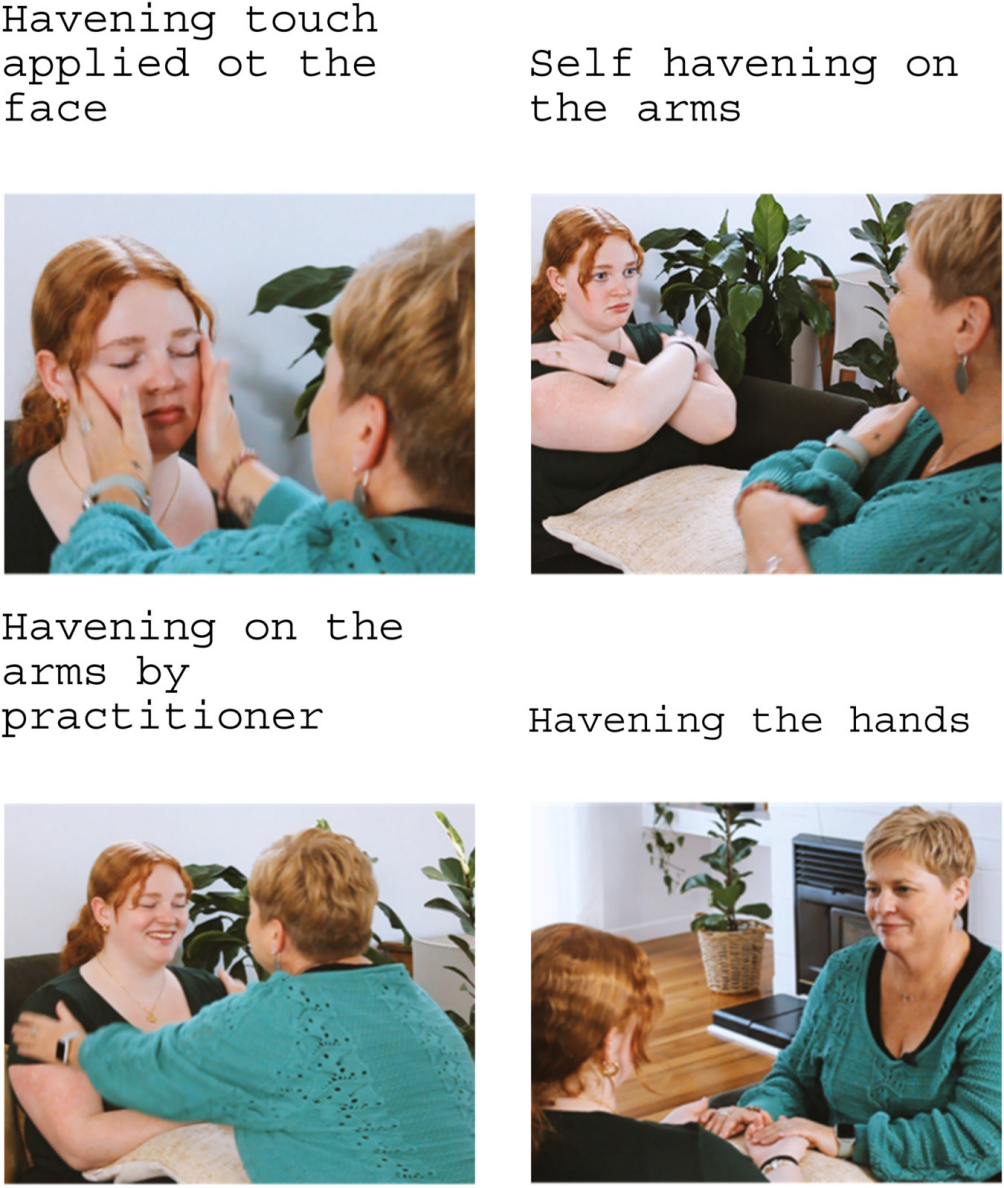
Demonstration of havening touch. Taken with permission from https://www.willowtherapy.co.nz.

Each round of HT with distractions (e.g., EH) is done for 5–7 min, corresponding with the length of time the calcium channels remain open between synapses. The woman then returns to the memory/emotion/belief and considers her SUD score and how the memory is presenting itself. The SUD score almost always reduces, and the memory tends to become more distant. Often the woman will feel an emotional disconnection from the memory, sometimes feeling that she is no longer present within the event. Additional HT may be used to reduce any negative emotions or self-belief remaining, and to strengthen resolve. The woman usually reports feeling “light” and relaxed on completion of the session, and the therapeutic effects are immediate. Each HT session ends with a debrief; the woman is asked to recall the memory again and CASE (Cognitive, Autonomic, Somatosensory, Emotional) is again considered to ensure that depotentiation has occurred. Figure 4 presents a series of four pictures demonstrating Havening Touch within a session; notice the self-havening touch and the practitioner provided havening touch. Written informed consent for this publication was obtained from the individuals in these images.

## 6 Impact of havening on perinatal mental health

As has been shown through the process of depotentiation, the emotional charge associated with distressing memories, such as a traumatic birth, can be reduced, allowing individuals to recall past experiences without experiencing the same level of stress, fear, anxiety, or anger. In the context of perinatal mental health, this means that individuals who have experienced birth trauma, anxiety, perinatal depression, or fear of birth can process these emotions in a way that diminishes their intensity, fostering greater emotional stability and resilience without impairing the mother-baby bond. As described in the introduction, traumatic birth experiences can result in morbidity. This can manifest as fear of subsequent births, anxiety and depression, a disruption in breastfeeding and the mother-infant relationship, and may even result in intergenerational trauma (48). Furthermore, a traumatic birth is also associated with an increased risk of self-harm, particularly for women experiencing postnatal depression (49, 50). Therefore, we contend that depotentiating AMPA receptor coded trauma is crucial for perinatal mental wellbeing and resilience.

Youngson (13) conducted a pilot research trial using HT with 29 participants who had experienced birth as traumatic and were experiencing intrusive symptoms of traumatic stress, some up to 20 years following birth. Youngson used the Impact of Events Scale (IES) to measure the severity of traumatic stress symptoms and found that 22 out of the 29 participants had an IES score indicating post-traumatic stress disorder (a score of 33 or above). Participants received individualised programmes of HT tailored to meet their needs. Findings show a 75% reduction in Post Traumatic Stress Disorder (PTSD) symptoms, with the average IES score reducing from 40 prior to Havening, to a score of 13 seven days after, and to a score of only 10, thirty days after Havening. Remarkably, 80% of the 21 participants with an IES score of 45 or less experienced rapid relief of PTSD symptoms after only a single HT session. Similarly, the impact of a single HT session on feelings of distress have been found in other empirical work on HT (42). Although Youngson's pilot study provides some encouraging evidence of how HT have a psycho-emotional health benefit during the perinatal period, more empirical research is needed.

In the following section we present four case studies to illustrate the impact HT can have on perinatal psycho-emotional health. Written informed consent was obtained from the individuals providing these case studies for use in this publication. Pseudonyms have been used to protect anonymity. See Table 5 for abbreviations and acronyms.

## 7 Case studies

### 7.1 CASE STUDY one: birth trauma. (Three HT sessions.)

Daisy was a 30-year-old woman, 25 weeks pregnant with her second baby, who presented with episodes of frequently recalling and crying over her first birth, a C-section. The most vivid and traumatic moment for her was when the doctor refused to honour her request to place the baby on her chest. Daisy was asked to recall the distressing moment and SUDs were calibrated at 9. Several rounds of distractions were used until SUDs was 2. Then RH was applied, where the midwife/Havener took the role of the doctor and asked for forgiveness for her lack of compassion, explaining that it was unintended. Daisy accepted her apology. Then OH was applied, where Daisy was invited to reimagine exactly how she would have liked the scene to have played out. Daisy sees the doctor kindly speaking to her and placing the baby on her chest. She was asked to imagine the feel of the warm and wet baby against her skin and to imagine smelling her baby. Daisy expressed feeling incredibly happy and was asked to repeat the word “happy” several times.

When asked to recall her C-section experience, she said: “Every time I think of my first birth, the experience is the opposite. All I can see is myself enjoying holding my baby.” Daisy had a successful VBAC that she described as empowering and everything she hoped for.

### 7.2 CASE STUDY two: prenatal depression and birth trauma (Three HT sessions.)

Megan was a 36-year-old woman, 12 weeks pregnant with her third baby. She presented with severe prenatal depression with intrusive thoughts and suicidal ideation. She was unable to get out of bed and described a constant feeling of incredible sadness and “heaviness.” Although this was a carefully planned and wanted pregnancy, she couldn't help having negative thoughts, such as: “This is the dumbest idea. You can't even take care of two kids, how are you going to take care of a third?” She often thought that if she died, it would “save everyone the trouble”, as she put it. As a social worker herself, she was able to recognize herself as clinically depressed.

Megan's first birth was very traumatic for her, and never sought therapy around her experience. Her son was born prematurely, and was given a slim chance of survival. She recalled the several months that she and her husband took turns watching over him in the hospital, often not knowing if he would make it. Her SUDs score when recalling that period was a 10, and several rounds of distractions were applied until the SUDs were 2. The word “safe” was used as an AF. Megan began to reveal that even though her second birth was uneventful and even powerful, she had suppressed the memory of the first birth until now, and believed that this prenatal depression might be linked to that first birth. She was surprised but also relieved by this revelation.

In subsequent HT sessions, Havening touch was applied while she explored her feelings around her first birth. When she recalled fear, distractions were used until SUDs went down to zero. When sadness came up, she was asked what the opposite of sadness would be. She said “happy,” and she was asked to alternately repeat the words “sad” and “happy.” (Opposite Havening). Although she was feeling a little skeptical, she followed the instructions. To her surprise, Megan eventually felt what she described as light and joyful. As AF, she was invited to repeat “light and joyful.”

Megan's mental state remained positive during her pregnancy and six weeks postpartum. A referral to other forms of therapy was deemed unnecessary. As Megan was approached for permission to use her case 6 years later, she reported that her depression did not return after her HT sessions.

### 7.3 CASE STUDY three: birth trauma and tokophobia

Sunny was a 31-year-old woman at seven months postpartum and presented with birth trauma after a c-section. She found herself constantly crying and feeling on “high alert”. She had also been unable to drive past the hospital where the birth took place. She expressed feelings of guilt, anxiety, and lacking in confidence as a mother. In addition, she recently found out that she was pregnant and was experiencing feelings of dread around her pregnancy and birth.

Sunny experienced a panic attack as she was wheeled into the theatre for her c-section. She connected it to a similar childhood event that was incredibly traumatic, where she was also being wheeled into surgery alone, separated from her mother, and felt terrified as she saw the surgical instruments in the theatre and the mask was pressed down on her face. She was put under general anesthesia for the c-section because of her emotional state. On waking up, she felt emotionally numb and was “detached” when she first met her baby. The numbness lasted a few weeks, followed by feelings of anxiety and flashbacks to the moments before the c-section. As she was speaking, her SUDs were calibrated at 10. Event Havening was immediately performed with four rounds of distractions until SUDs went down to 0. In the debrief, Sunny expressed feeling relaxed and calm and able to recall her birth experience without distress.

Transpirational Havening (HT) was done on her remaining feelings of anger and guilt, after which she described a dense fog being lifted from her. When asked to recall her childhood surgical experience, Sunny was surprised that some of the “emotional heat” had already gone from this. Outcome Havening was used to help her change the outcome of the childhood surgery into a more positive experience. She imagined her mother taking her into the theatre and holding her hand, instead of being taken in alone feeling vulnerable and scared. In the debrief, Sunny expressed a feeling of peace and safety in place of vulnerability and panic. Sunny reported post Havening that she finally bonded with her baby and felt more confident in her mothering and believed that her son was more relaxed because of her new inner calm. She also reported feeling more positive in her current pregnancy.

### 7.4 CASE STUDY four: tokophobia

Mia was a 27-year-old woman at 33 weeks, pregnant for the first time, who presented with anxiety about her upcoming birth. She was concerned she might feel out of control; her personal agency potentially being compromised during childbirth. She felt so vulnerable and powerless to the labour and birth process that she considered having an elective c-section, but was also worried that surgery would create its own problems.

When asked if there had been experiences in her past where she may have felt similarly vulnerable and powerless, Mia disclosed that although she had a relatively happy childhood, her parents had been very strict. As a punishment, she was often shut in her wardrobe for what felt like a long period of time. One distressful memory stood out when she heard the front door of the house close and thought her parents were leaving. As Mia was speaking, SUDs were calibrated at 7. Event Havening (EH) applied using four rounds of distraction until SUDs was at 0. Following that session, Mia expressed feeling “emotionally light” and that when she recalled her experiences, she felt she was “a distance away, and the picture was blurred”. It was as though the event was happening to another girl, not her.

Next, Mia wanted to work on her low self-esteem, especially when it came to successfully completing something. As a child, she frequently doubted her abilities and worried about failing. Aspirational Havening (AH) was applied, asking her to focus on the emotions that she wanted to feel about herself. Outcome Havening was used to visualize the labour and birth that Mia wanted, paying particular attention to her ability to make decisions for herself, and her own inner strength. She also visualized having her midwife for support, as well as the people she chose to be present. Mia was taught to self-haven to continue this work daily to strengthen her resolve at home. Post havening, Mia reported feeling much less anxious about her upcoming birth and feeling confident about birthing physiologically. Subsequently, Mia birthed physiologically reporting that her birth was a positive and empowering experience.

## 7 Discussion

In the case studies we have shown how Havening reduces the emotional impact of distressing experiences, allowing the woman to recall past challenges without being overwhelmed by fear, anxiety, or distress. Moreover, by reinforcing her adaptive coping mechanisms, HT helps the woman to reprocess past experiences in a way that fosters new perspectives, releasing her from the cycle of fear or distress. She is then able to develop a more empowered and constructive outlook on her past and current childbirth experiences. Ultimately, HT can help to release the woman from the weight of her past traumatic experiences or emotions, along with the ripple effects of this trauma. The simplicity and accessibility of HT enable a woman to practice self-care techniques independently, reinforcing a sense of control over her emotional state and experience, empowering self-healing through the self-directed ability for ongoing self-havening. The four anecdotal case studies from practice support possible benefits that Havening can have during the perinatal period, illuminating the ripple effects that unresolved trauma and fear can have on maternal wellbeing, birth outcomes, and the transition to parenthood.

The case studies show that there is often more depth to the trauma/emotion/belief than is initially presented and understood, the unfolding nature of this therapeutic process, and how Havening practitioners safely support this exploration and resolve. Havening practitioners are trained to listen very carefully not just to the words that are spoken but are highly attuned to what is evident within the person's words, and what is expressed physiologically. Using CASE (Cognitive, Autonomic, Somatosensory, Emotions) in addition to SUDs helps practitioners to holistically assess the effects of the trauma on the person's emotional and physiological wellbeing. CASE is assessed at the start of the HT session, used as it unfolds, and is also assessed on completion of the session to ensure that depotentiation has occurred.

The case studies provide some appreciation of the careful history taking required, an incredibly important part of Havening. It is crucial that practitioners are always attuned to what could be underpinning or influencing the person's traumatic experience/emotions. This is particularly relevant in perinatal Havening, due to the significance that trauma – past and present, could have for the woman as she travels along the childbirth continuum. According to the founders of Havening Techniques, Ruden and Ruden “history taking is the key to removing the unwanted traumatically encoded pathway” (51 p. 128). Adverse Childhood Events (ACEs) increase a person's vulnerability for traumatization (14). Ruden (14) advocates for always looking beyond the trauma that is initially presented and seeking earlier life events that may have predisposed the landscape of the brain to vulnerability. In our Havening practices we, the three authors, have found that ACEs are a common occurrence with women presenting with trauma during the perinatal period, both representing trauma in themselves, and underpinning later traumatization.

The case studies reveal how HT sessions provide relief in psycho-emotional distress in the perinatal domain, yet it remains unclear if HT would as be effective for perinatal related physical pain. A 2018 study explored surgical pain and the use of HT, the findings did not indicate reduced surgical pain or less use of pain relief medication in the short term (34). This study was not focused on perinatal surgical pain and further empirical work is required to establish if HT can be a useful adjunct to care in the context of perinatal related physical pain. Hypothetically, researchers have suggested that HT stimulation of the brain's intrinsic potential to enable psychophysiological resilience and improve wellbeing with a non-pharmaceutical intervention with no known side-effects is intriguing (33). This would be a welcome addition to therapeutic approaches in the perinatal context wherein pharmaceutical use could be reduced. The authors have certainly encountered relief of physical perinatal related physical pain in HT sessions when addressing psycho-emotional distress. Therefore, the link between psychological distress and physical pain in the context of perinatal HT necessitates further examination.

Reflecting on the four case studies, three areas are evident and are important to emphasise in terms of HT sessions: (a) Havening is relational and the comportment of the Havening practitioner is crucial, (b) Havening is intimate, it involves touch, and (c) Havening works on events that are personally challenging.

It is important to highlight the therapeutic relationship between the Havening practitioner and the client. Creating, being, and holding a safe place for people to be and feel nurtured, seen, heard, and understood is fundamental to HT being efficacious. People must feel safe to share their experiences and to freely and authentically express their emotions. During the HT process the person moves into a very relaxed state, and many come to HT sessions having been traumatized by others including health care providers, so feeling safe to revisit their thoughts and experiences, and to relax into the evolving therapeutic process whilst feeling metaphorically held, is incredibly important.

Not all people having Havening feel comfortable, or able, to verbally share the details of their traumatic experiences/emotions/beliefs. This is by no means a barrier to Havening being efficacious provided that the events/feelings/beliefs can be brought to the forefront of the mind and silently revisited by the person at the start of the Havening session, and when required as the session unfolds. This is known as content-free Havening. Not having to share these details can be of significant benefit if the person feels so traumatized that they simply cannot verbalize their experiences or feelings, or they feel embarrassment, guilt, or shame. This can be particularly helpful when working with teenagers.

Similarly, not all people having Havening are comfortable with touch. However, Havening touch can be done by the practitioner, through self-havening, or a support person whilst the Havening practitioner facilitates the session. These options are always offered at the start of each session, and a check-in is done to ensure that the person is comfortable with the practitioner applying the touch if this was the option chosen (see images in Figure 4). All options effectively generate the desired electroceutical changes (i.e., oscillations in the delta frequency and neurochemicals). Should the person apply havening touch themselves they need to be able to do this continually, and this can sometimes be challenging and requires lots of encouragement when they begin to feel intense relaxation. In our practices most women prefer Havening practitioners to apply the touch. If the woman feels comfortable with this, feeling physically held can be therapeutic and this can help them to relax into the process.

## 8 Conclusion

The etiology of the word trauma from the Ancient Greek word “*τρ*ά*υμα*” means “wound” or “hurt”. Although associated with physical injuries does encompass emotional and psychological wounding. Furthermore, it can be construed that the physiological impact of psycho-emotional trauma on the amygdala is akin to physical wounding. This article has focused on this wounding in the perinatal mental health domain highlighting the psycho-emotional wounding or deep hurting (past and present), and spiritual distress (i.e., loss of meaning and purpose) of some women as they traverse the perinatal period. The modality of HT has been presented as a potential healing balm to this wounding.

In this theoretical discussion and case study presentation we have shown how in the perinatal period, the psycho-sensory therapy of HT has benefits which anecdotally translate into improved maternal well-being, reduced vulnerability to stress and anxiety, and a greater capacity to bond with the baby in a calm, connected manner. By addressing amygdala-driven emotional dysregulation, HT has the potential to alleviate symptoms of perinatal anxiety, depression, PTSD, and phobias. Women who experience traumatic births or fear surrounding childbirth may find relief through HT's ability to reframe distressing memories, discover new meanings related to traumatic experiences and reduce physiological stress responses. Additionally, its purported capacity to enhance oxytocin release supports maternal-infant bonding, fostering a more positive postpartum experience for the mother-baby dyad. As either a stand-alone modality or an adjunct to traditional mental health treatments, HT offers a gentle, empowering, and non-invasive accessible healing method to enhance emotional and spiritual well-being during the perinatal period.

Havening Technique's ability to modulate amygdala function and reduce distressing emotional memories presents a promising adjunct to traditional perinatal mental health interventions. Dr. Ruden's theoretical framework highlights the neurobiological basis for its effectiveness, emphasizing the role of delta waves in depotentiating traumatic memories. Whilst theoretical reasoning and anecdotal practice-based experiences are compelling, it is important that the use of and impact of HT during the perinatal period is supported with more empirical studies. Further research is needed to establish HT's long-term efficacy and integration into perinatal care frameworks. Nonetheless, preliminary evidence and anecdotal experiences suggest that HT may serve as a valuable tool in fostering mental and spiritual wellbeing, and psycho-emotional resilience during the perinatal journey.

## Data Availability

The original contributions presented in the study are included in the article/Supplementary Material, further inquiries can be directed to the corresponding author.
